# Novel genome-wide DNA methylation profiling reveals distinct epigenetic landscape, prognostic model and cellular composition of early-stage lung adenocarcinoma

**DOI:** 10.1186/s12967-024-05146-2

**Published:** 2024-05-06

**Authors:** Junwen Gan, Meng Huang, Weishi Wang, Guining Fu, Mingyuan Hu, Hongcheng Zhong, Xin Ye, Qingdong Cao

**Affiliations:** 1 Department of Thoracic Surgery, The Fifth Affiliated Hospital of Sun Yat-Sen University, Zhuhai, 519000 Guangdong China; 2 Zhuhai Sanmed Biotech Ltd, No. 266 Tongchang Road, Xiang Zhou District, Zhuhai, Guangdong P. R. China; 3 Joint Research Center of Liquid Biopsy in Guangdong, Hong Kong, and Macao, Zhuhai, China

**Keywords:** Early-stage lung adenocarcinoma, DNA methylation, Enzymatic methyl sequencing (EM-seq), Epigenetic landscape, Prognostic model

## Abstract

**Background:**

Lung adenocarcinoma (LUAD) has been a leading cause of cancer-related mortality worldwide. Early intervention can significantly improve prognosis. DNA methylation could occur in the early stage of tumor. Comprehensive understanding the epigenetic landscape of early-stage LUAD is crucial in understanding tumorigenesis.

**Methods:**

Enzymatic methyl sequencing (EM-seq) was performed on 23 tumors and paired normal tissue to reveal distinct epigenetic landscape, for compared with The Cancer Genome Atlas (TCGA) 450K methylation microarray data. Then, an integrative analysis was performed combined with TCGA LUAD RNA-seq data to identify significant differential methylated and expressed genes. Subsequently, the prognostic risk model was constructed and cellular composition was analyzed.

**Results:**

Methylome analysis of EM-seq comparing tumor and normal tissues identified 25 million cytosine-phosphate-guanine (CpG) sites and 30,187 differentially methylated regions (DMR) with a greater number of untraditional types. EM-seq identified a significantly higher number of CpG sites and DMRs compared to the 450K microarray. By integrating the differentially methylated genes (DMGs) with LUAD-related differentially expressed genes (DEGs) from the TCGA database, we constructed prognostic model based on six differentially methylated-expressed genes (MEGs) and verified our prognostic model in GSE13213 and GSE42127 dataset. Finally, cell deconvolution based on the in-house EM-seq methylation profile was used to estimate cellular composition of early-stage LUAD.

**Conclusions:**

This study firstly delves into novel pattern of epigenomic DNA methylation and provides a multidimensional analysis of the role of DNA methylation revealed by EM-seq in early-stage LUAD, providing distinctive insights into its potential epigenetic mechanisms.

**Supplementary Information:**

The online version contains supplementary material available at 10.1186/s12967-024-05146-2.

## Introduction

Lung cancer (LC) remains a prevalent cause of cancer incidence and mortality globally, accounting for 11.4% of all cancer diagnoses and 18% of total cancer-related deaths in 2020 [1]. Among the various pathological types of lung cancer, lung adenocarcinoma (LUAD) is the predominant type, representing approximately 85% of all cases [2]. While the overall 5-year survival rate for lung cancer in China remains below 30%, it can drop to 7% once the cancer progresses to advanced stage. Consequently, there is an urgent need for in-depth research into early-stage LUAD [3]. As the number of new cases of early-stage LUAD continues to rise, the identification of specific tumor biomarkers is crucial for improving diagnosis, treatment, and prognosis.

DNA methylation is one of the most common epigenetic modifications in humans. It mainly involves the modification of cytosine at the fifth carbon position by adding methyl groups to form 5-methylcytosine, which can be oxidized to form 5-hydroxymethylcytosine [4]. Dysregulation of DNA methylation has been linked to various malignant tumors [5–7]. In cancer, a global hypomethylation pattern is typically observed, leading to genomic instability and activation of silenced oncogenes [8, 9]. Additionally, promoter hypermethylation frequently results in the inactivation of tumor suppressor genes (TSGs), which is considered a major contributor to neoplastic transformation [10]. Notably, aberrant DNA methylation can occur at very early stage of tumors [8], suggesting that studying DNA methylation in early stage of tumors holds significant clinical translational potential for the future.

A novel DNA methylation detection technique, Enzymatic methyl sequencing (EM-seq), utilizes a combination of biological enzymes and chemical methods to achieve similar conversion to bisulfite treatment for methylation analysis without damaging the template DNA [11]. EM-seq offers several advantages, including improved coverage and a more even GC distribution, especially with low-amount input [12]. But EM-seq has been rarely applied in cancer such as LUAD.

In this study, we used EM-seq to identify more distinctive methylation signatures in early-stage LUAD patients compared to 450 K methylation microarray. Furthermore, we selected differentially methylation-expression associated genes (MEGs) to construct a prognostic risk model and utilized cell deconvolution algorithm to estimate cell composition in early-stage LUAD based on the in-house EM-seq results. These findings underscore the importance of DNA methylation in early-stage LUAD tumorigenesis and identify potential biomarkers for future research.

## Methods

### Patients and samples

Patients with LUAD that were treated at the fifth affiliated hospital of Sun Yat-Sen university, between October 2022 and December 2022 were selected for this study. We included LUAD patients according to the following criteria: age 18–75 years old (no pregnant or breastfeeding); patients were preliminarily identified as LUAD by CT examination; no received any therapy (radiotherapy, chemotherapy or immunotherapy) before. Patients who had respiratory infection within the last month, other malignancies or previous history of cancer, and received neoadjuvant therapy were excluded. All patients were pathologically confirmed with LUAD by at least two experienced pathologists according to The American Joint Committee on Cancer (AJCC) 8th Edition Staging System. Both lesion tissue and adjacent normal tissue at a distance of at least 5 cm away were taken simultaneously through surgery. The tissue size was 5*5* 5mm^3^. Information on characteristics including age, gender, tumor location, TNM stage and pathological differentiation was also obtained.

### EM-seq process

Genomic DNA was isolated from fresh-frozen tissue samples using the D Neasy Blood & Tissue Kit (Qiagen, Valencia, CA). The gDNA was sonicated into 300 bp fragments using Covaris M220. 50 ng sonicated gDNA were used to construct libraries with NEBNext EM-seq Kit (New England Biolabs, Ipswich. MA, USA). Then libraries were pair-end-sequenced on Illumina NOVA_S4-G-PE150 with 1% Phix control library added. The sequencing data was about 90Gb, equivalent to 30× average sequencing depth. There are two step conversion of the cytosines. The first step uses TET2 oxidation of 5methylcytosine that provides protection to the modified cytosines from conversion by apolipoprotein B mRNA editing enzyme catalytic deamination, so only cytosines but not 5-methylcytosines are converted to uracil. Ultimately, cytosines are sequenced as thymines and 5-methylcytosines are sequenced as cytosines.

### Methylation data processing

Per-CpG methylation level was defined as the percentage converted from Methylation Beta Value [13] representing the ratio between the methylated array intensity and total array intensity and falling between 0 (lower levels of methylation) and 1 (higher levels of methylation) for 450 K array data while the percentage of methylative reads to reads covered per CpG site for EM-seq data.

AS for EM-seq data, initial data analysis was performed as part of the standard snakePipes-WGBS pipeline. Paired-end reads were filtered as well as trimmed by Fastp (v0.20.0, parameters ‘-q 5 -l 30 -M 5’) and then mapped to the human genome (GRCh38_release93) using bwa-meth (v0.2.2). The PCR duplicates were marked with sambamba. Methylation ratioslevel were extractedcalculated via MethylDackel (v0.5.0, parameters ‘--mergeContext --maxVariantFrac 0.25 --minDepth 1’) for CpG positions in the reference genome. Metilene (v0.2.8) [14] with default settings was used to find differentially methylated regions (DMRs) by comparing tumor to paired-normal samples.

R-ChAMP(v2.26.0) [15] pipeline was used for the follow-up analyses about TCGA 450K data processing. First, probes that belong to non-CpGs, including SNPs [16], align to multiple locations, and located on X are Y chromosomes were filtered out with the champ.filter function. Then the filtered data were normalized with the BMIQ method for types I and II probe correction by the champ.norm function.

Considering the precision effect of sequencing depth on methylation level, CpG sites with median coverage less than 10X on EM-seq dataset were not shown in the boxplot of methylation level. Note that all CpG sites on sex chromosomes were filtered out. Density of CpGs/DMRs on chromosome was defined as counts of CpGs/DMRs divided by length of chromosomes extracted from genome reference by using Samtools(vl.9).

### Identification of DMR and epigenomics annotation analysis

Identified as described upon, EM-seq DMRs were further selected by filters ‘MeanDiff (Mean difference of methylation levels of CpG sites in a DMR) ≥ 10% and adjP < 0.05’ and defined as significant DMRs. 450 K DMRs were identified by using Bumphunter [17] method with default parameters setting. Significant DMRs were considered as DMRs conformed to filters ‘Value ≥ 1 and adjP < 0.05’.

Gene feature annotation of the identified DMRs was performed against Ensembl genomic annotations using the R package annotatr(v1.26.0) [18]. Multiple Annotations of DMRs were deduplicated by a custom script following the priority principle: ‘Promoter’>’1to5kb’> ‘5UTR’>’ firstexon’>’ exon’>’ intron’>’ intronexonboundary/exonintronboundary’>’ intergenic’.

Genic annotations include 1-5Kb upstream of the TSS, the promoter (< 1Kb upstream of the TSS), 5’UTR, first exons, exons, introns, CDS, 3’UTR, and intergenic regions (the intergenic regions exclude the previous list of annotations).

### Differential expression analysis and MEG selection

A total of 586 samples of RNA-sequencing data from TCGA cohort were enrolled in this study (excluding Formalin-Fixed Paraffin-Embedded (FFPE) and only remain Primary Tumor and Solid Tissue Normal samples). The DESeq2 was employed for the analysis of expression of DEGs based on raw read counts. Those genes that had a false discovery rate (FDR)<0.05 and the absolute value of log2 fold change difference (|log2 (FC)|) ≥ 1 were considered to be differentially expressed.

DMGs were identified by Gene feature annotation of the identified DMR. The intersection of EM-seq significant DMGs and TCGA DEGs was selected as MEG.

### GO and KEGG enrichment analysis of MEGs

To annotate the biological function of each MEG, we performed Gene Ontology (GO) and Kyoto Encyclopedia of Genes and Genomes (KEGG) enrichment analysis by clusterProfiler [19]. The hypergeometric distribution was used to test the significance of functional categories in MEGs, and the KEGG/GO term satisfying a P value ≤ 0.05 was defined as significant in the KEGG/GO term enriched by MEGs.

### Survival analysis and construction of risk model

Row counts of DEGs were then converted to transcripts per million for model training and least absolute shrinkage and selection operator (LASSO) regression analysis was utilized to detect survival-related MEGs. Further, Risk signatures were generated through the linear combination of regression coefficient values from multivariate Cox regression model coefficient values and gene expression levels as follows:

$$\begin{gathered}Risk\,Score = \hfill \\\,\,\,\,\,\,\,\,\,\,\,\,\,\,\sum {i\,Coefficient(mRNAi) \times Expression(mRNAi)} \hfill \\ \end{gathered}$$

Median risk score values were utilized to separate patients into low- and high-risk groups, and risk signature efficiency was evaluated via the Kaplan-Meier(K-M) approach. Harrell’s concordance index (C-index) and time-dependent receiver operating characteristic (tROC) curve analysis within 1, 3 and 5 years were used to evaluate the predictive accuracy of the prognostic model. Then, the performance of the prognostic model constructed by the TCGA training set was validated in the GSE42127 and GSE13213 cohorts via a similar approach.

### Cell deconvolution analysis

Cell deconvolution on EM-seq data was analyzed using the UXM [20] algorithm, a computational fragment-level deconvolution algorithm for DNA methylation sequencing data and used the top 25 markers defined for each cell type (a total of 1,246 markers) to study methylomes obtained from composite tissue samples.

### Immunohistochemistry (IHC)

Formalin-fixed, paraffin-embedded LUAD and normal lung tissue specimens were sectioned into 5 μm thick slices. All sections were dewaxed with xylene, hydrated with gradient alcohol, washed with phosphate-buffered saline. Subsequently, the sections were boiled in a pressure cooker at 100℃ for 10 min. Ethylenediaminetetraacetic acid (EDTA) buffer (pH = 9.0) was suitable for CD3, CD19 and CD56 staining, while sodium citrate buffer (pH = 6.0) was suitable for CD31 and pulmonary-associated surfactant protein C (SP-C) staining. The tissue samples were blocked with 3% H2O2 solution for 10 min at room temperature to eliminate the endogenous peroxidase activity and were blocked with 1% bovine serum albumin (BSA, Neobioscience, NBS-BSA) for 1 h at room temperature. Next, the tissue samples were incubated with CD3 (IR020, LBP), CD19 (IR354, LBP), CD31 (IM030, LBP), CD56 (IM040, LBP) or SP-C (1:200 dilution, DF6647-50, Affinity Biosciences) antibodies overnight at 4℃. After the sections were incubated with the secondary antibody (PV-6000, ZSGB-BIO) for 1 h at room temperature, DAB (ZLI-9017, ZSGB-BIO) was used as the chromogen. Finally, the sections were counterstained with hematoxylin and mounted. All sections were scanned as images by an automatic digital slide scanner (3D-HISTECH Digital Pathology Company, Hungary). Cells with positive staining was defined as those with a yellowish–brown staining of the cytoplasm or cell membrane. Two independent observers determined staining results and discussed them to reach a consensus in contradictory cases.

### Statistical analysis

All statistical analyses were conducted using R software (v4.2). The Wilcoxon test was utilized to compare the median methylation levels and proportions of cell-types between tumor and paired normal tissue samples. LASSO Cox regression analyses was performed to construct and evaluate the prognostic predicated models using “glmnet(v4.1-7)” [21, 22] and “survival(v3.5-5) [23]” packages of the R software. ROC curve analysis was performed to predict the OS of LUAD patients using the “timeROC(v0.4) [24]” package in the R. The OS between the two clusters was analyzed by Kaplan-Meier analysis with the log-rank test. A P value less than 0.05 was considered statistically significant.

## Results

### Comprehensive collection of CpG sites in LUAD by EM-seq comparing with 450K microarray

**Fig. 1:**
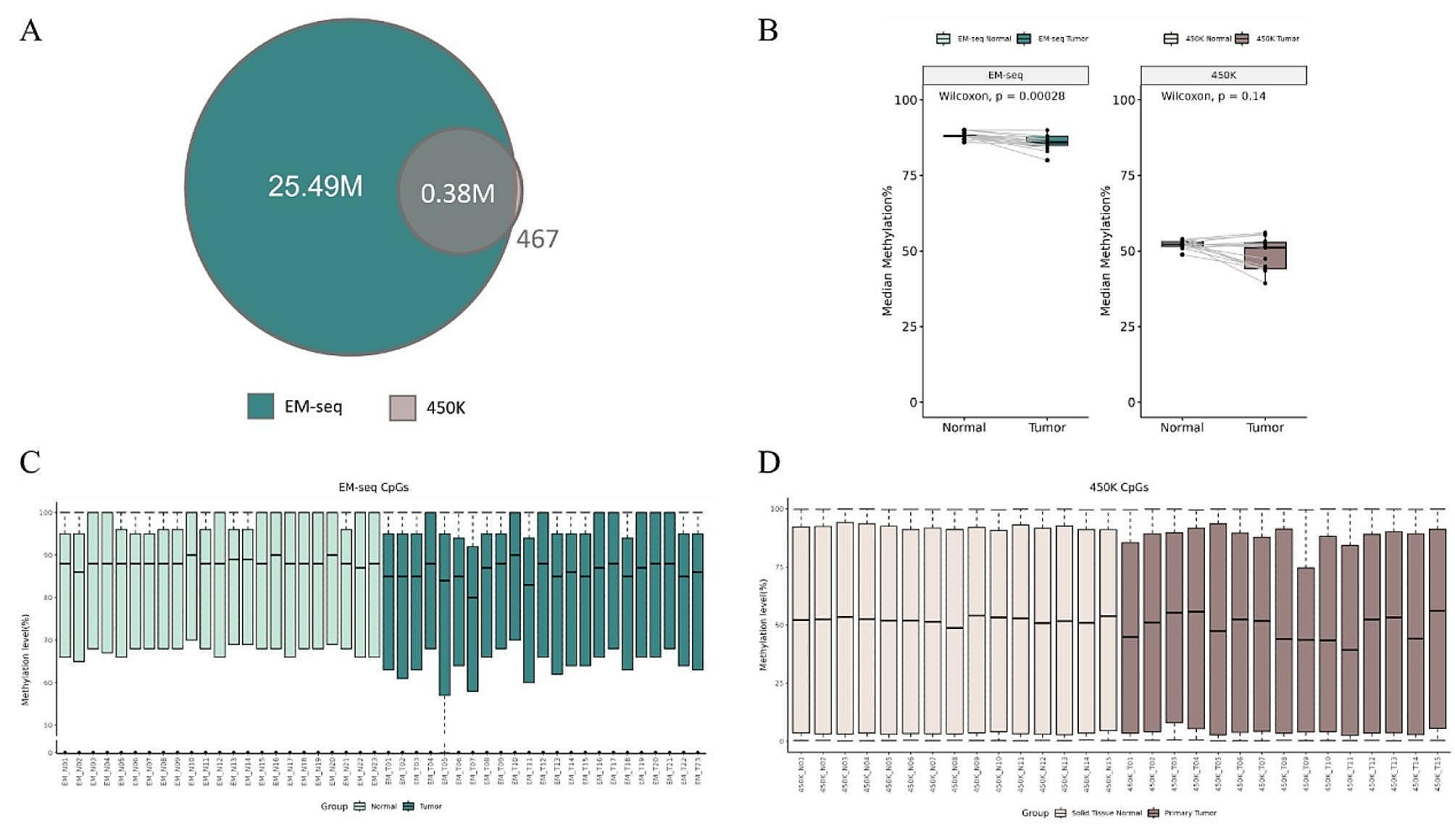
Global DNA methylation profile in early-stage LUAD. **(A)** Venn diagram displaying CpG sites (filtered for sex chromosomes and sequencing depth) measured by EM-seq(green) and 450K(brown). **(B)** Comparison of median CpG methylation levels between tumor and normal samples in EM-seq(left) and 450K(right). **(C)** Box plots displaying the methylation levels of each normal (light green) and tumor (bottle green) sample in EM-seq.  **(D)** Box plots displaying the methylation levels of each normal (pink) and tumor (café) sample in 450K. *P* values computed from Wilcoxon tests. EM-seq, *n* = 23; 450K, *n* = 15

All tumor and paired normal tissue samples from 23 LUAD patients (22 stage I + 1 stage III) were analyzed using EM-seq, resulting in the detection of a total of 28 million CpGs. After some conditions filtering (CpG sites on sex chromosomes, NA = 0%, median sequencing depth < 10%), approximately 25 million CpGs were identified. Additionally, we selected 450K methylation microarray data (filtering NA = 0%) of 15 LUAD patients (12 stage I + 1 stage III + 1 stage IV) from TCGA database for comparison (Table 1). Notably, the number of CpGs detected by 450K microarray was only 0.38 million, which was almost completely covered by EM-seq (Fig. 1A). To present a more detailed and comprehensive methylation profile for each sample, we evaluated the per-CpG methylation level in each sample and displayed the upper quartile, median, and lower quartile of methylation levels (%). Whole-genome hypomethylation was observed in tumor tissues based on EM-seq and 450K microarray data (Fig. 1B). Methylation level of EM-seq samples was mostly over 60%, with the upper and lower quartiles ranging from 60 to 95% and the median at approximately 85%. In contrast, the upper and lower quartiles of 450K microarray were approximately 5–90%, with a median of about 50%, which was lower than EM-seq. Furthermore, it was found that the methylation level of CpG sites covered by 450K microarray on EM-seq samples was lower than the methylation level of EM-seq CpGs on EM-seq samples (Supplementary Fig. S1), indicating that the difference in methylation levels measured by the two methods may be attributed to their covered CpG sites distributed in different gene feature regions. In both EM-seq and 450K microarray, the methylation levels were similar between each normal tissues but varied significantly between each tumor tissues (Fig. 1C and D). Moreover, it appeared that the larger the tumor dimension diameter, the greater the difference in methylation levels between tumor and normal samples (Supplementary: Table S2).

**Table 1:** Basic demographics of patients enrolled in EM-seq and 450K

	EM-seq	450K
*n* (Pairs)	23	15
**Age (%)**		
< 70	22(96%)	10(67%)
≥ 70	1(4%)	5(33%)
**Gender (%)**		
Female	22(96%)	10(67%)
Male	1(4%)	5(33%)
**Stage (%)**		
Stage I	22(96%)	12(80%)
Stage III	1(4%)	1(7%)
Stage IV	0	2(13%)

**Fig. 2:**
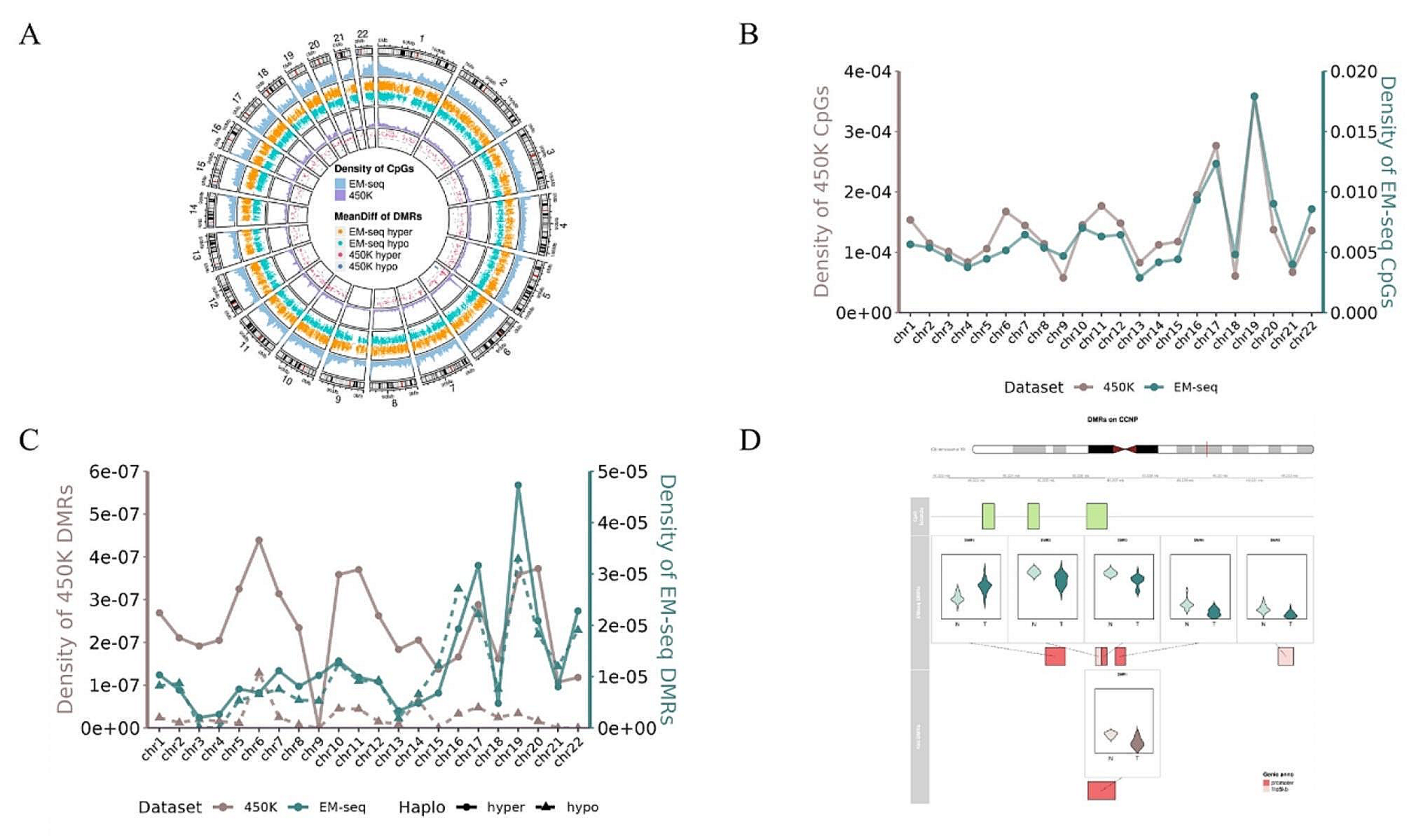
Distribution of DMRs across the whole genome. **(A)** Circos diagram of the distribution of CpGs and DMRs at the chromosome level. 6 color bars in the heatmap from outside to inside represent the following: EM-seq CpGs density (indigo), EM-seq hyper DMRs (organe), EM-seq hypo DMRs (green), 450K CpGs density (purple), 450K hyper DMRs (red) and 450K hypo DMRs (blue). **(B)** Linear graph of density of EM-seq(green) and 450K(brown) CpGs on each chromosome. **(C)** Linear graph of density of EM-seq(green) and 450K(brown) DMRs on each chromosome. Circular symbolize hyper DMR and arrow symbolize hypo DMR. **(D)** Distribution of specific CpG islands, EM-seq DMRs and 450K DMRs of CCNP on chromosome 19. Gene annotation: promoter region (red);1 to 5 kb region(pink)

We aimed to determine if there was a difference in the distribution of CpGs on each chromosome. So, we defined the density of CpGs as the number of CpGs on each chromosome divided by the length of each chromosome (in Mb). The density of CpGs from EM-seq on each chromosome was significantly higher than that of 450K microarray. However, their distribution patterns on chromosomes were very similar (Fig. 2A and B).

### Overall epigenetic profile of DMRs in EM-seq and 450K

Considering the spatial relationship of DNA methylation and the possibility that methylation changes in larger regions may have more significant biological functions, we focused on differentially methylated regions (DMRs). A total of 30,187 DMRs were identified in EM-seq, including 16,486 Hyper DMRs (54.6%) and 13,701 Hypo DMRs (45.4%). In contrast, only 787 DMRs were identified using 450 K microarray under similar filtration, significantly fewer than EM-seq, with 709 for Hyper DMRs (90%) and 78 for Hypo DMRs (10%), indicating that 450K microarray sequencing mainly detected hypermethylation DMRs. We also defined the density of DMRs as the number of DMRs on each chromosome divided by the length of each chromosome (in Mb). The DMR density of EM-seq was much higher than 450K microarray on each chromosome, but their distribution patterns of DMRs were notably different. In EM-seq, the distribution of Hyper DMRs and Hypo DMRs almost coincided, while in 450K microarray, the density of Hyper DMRs was significantly higher. Interestingly, although some 450K microarray CpGs did exist on chromosome 9, neither hyper nor hypo DMRs were identified on this chromosome. Furthermore, we found that DMRs were completely absent in the centromere (Fig. 2A and C).

From a whole-genome perspective, it was evident that EM-seq was able to demonstrate a greater quantity and variety of DMRs compared to 450K microarray, which was consistent with the specific gene. For instance, atypical cyclin P (CCNP), encoded on chromosome 19, exemplifies this difference. While 450K microarray only detected one hypomethylated DMR in its promoter region, EM-seq identified multiple DMRs with distinct patterns and levels of methylation. These DMRs were distributed across various regions, including promoter region and 1 to 5 kb region. These results highlight the ability of EM-seq to provide a comprehensive view of DNA methylation patterns (Fig. 2D).

### Identification and functional analysis of MEGs in EM-seq and 450K microarray

**Fig. 3:**
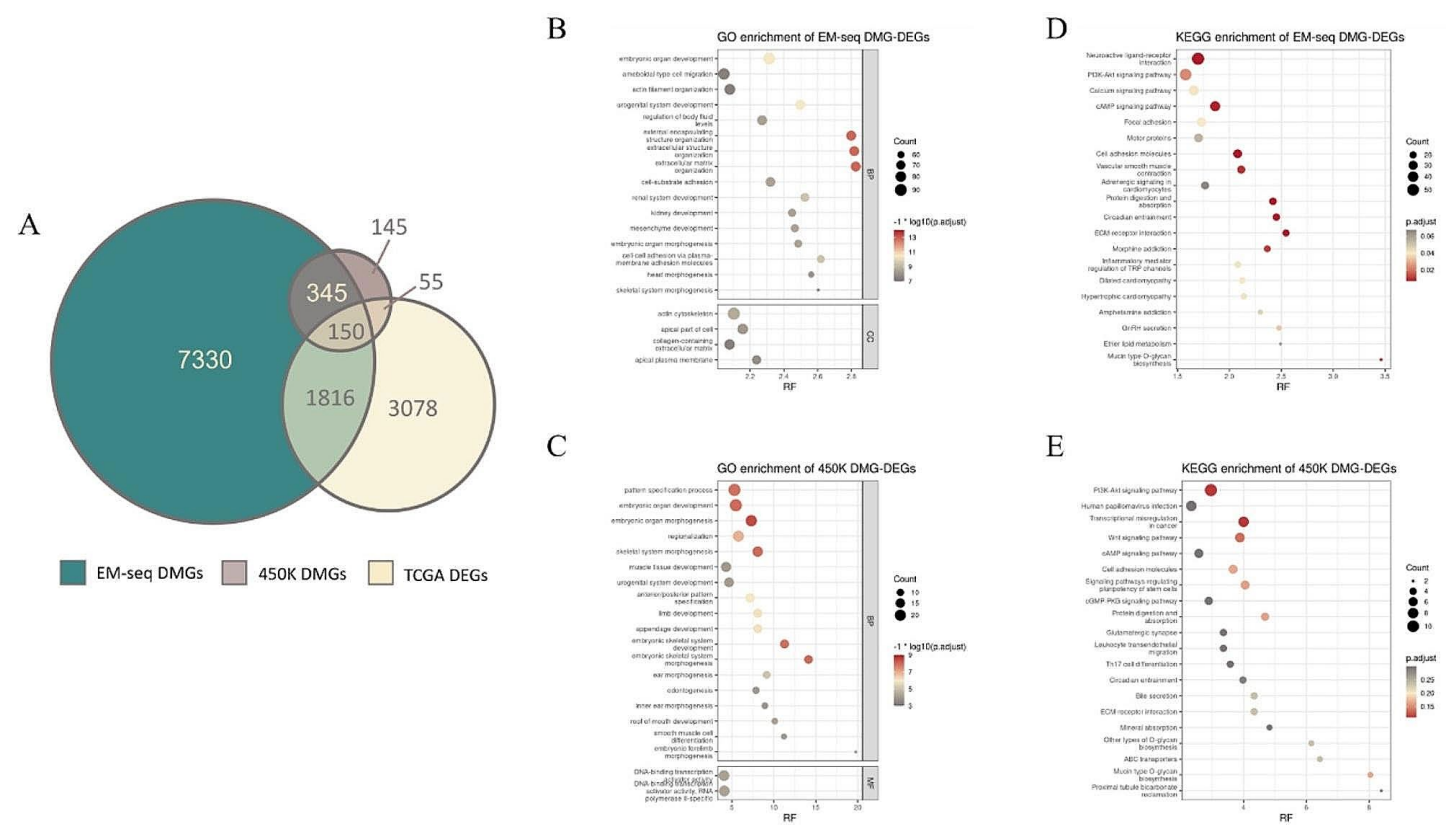
Biological functions of DMGs in EM-seq and 450K. **(A)** Venn diagram displaying the overlap between EM-seq(green)/450K(brown) DMGs and DEGs(yellow). GO analysis for 450K MEG-DEGs **(B)** and EM-seq MEG-DEGs **(C)**. KEGG analysis for 450K MEG-DEGs **(D)** and EM-seq MEG-DEGs **(E)**

After DMR-related gene annotation, we obtained 9641 and 695 DMGs from EM-seq and 450K microarray, respectively. In addition, we selected 5099 DEGs in the TCGA LUAD database, comprising 3171 up-regulated genes and 1928 down-regulated genes. The number of differentially methylated-expressed genes (MEGs) defined as overlapping genes between the DMG and DEG sets were 1966 for EM-seq and 205 for 450K microarray (Fig. 3A). To gain insight into the potential biological function and signaling pathways of methylation in early-stage LUAD, we conducted GO and KEGG pathway analyses of MEGs. In the case of EM-seq, the GO enrichment results showed that genes were enriched in embryonic biological processes including pattern specification process, embryonic organ development, and embryonic organ morphogenesis (Fig. 3B). The KEGG enrichment result indicated that methylated genes from EM-seq were significantly enriched in Neuroactive ligand-receptor interaction, Phosphatidylinositide 3-kinases (PI3K)-Akt signaling pathway, and cyclic AMP (cAmp) signaling pathway (all *p* < 0.05) (Fig. 3C). For 450K microarray, the GO enrichment results also showed enrichment in embryonic biological processes, including embryonic organ development and ameboidal-type cell migration (Fig. 3D). The KEGG enrichment result indicated that methylated genes from 450K microarray were significantly enriched in the PI3K-Akt signal pathway, human papillomavirus infection, and Transcriptional misregulation in cancer (all *p* > 0.05) (Fig. 3E).

### Distribution of DMRs and corresponding MEGs by EM-seq and 450K microarray across the whole genome

The difference of distribution of CpG in genome between EM-seq and 450K microarray was obvious (Supplementary Fig. S2). Given the substantial number of DMRs we obtained, we were interested in determining whether all DMRs were negatively regulated its expression, and whether the expression patterns of DMRs at different genomic locations were similar. The DMRs and corresponding DEGs were divided into four types: “Hyper-Up” for the hypermethylated and upregulated genes; “Hyper-Down” for the hypermethylated and downregulated genes; “Hypo-Up” for the hypomethylated and upregulated genes and “Hypo-Down” for the hypomethylated and downregulated genes. The distribution of EM-seq DMRs in all types were completely different from 450K microarray. Most of 450K microarray DMRs were mostly distributed in the promoter region (about 60-80%) (Supplementary Fig. S3). In EM-seq, except for about 20% DMRs distributing in promoter region, large proportion of DMRs were distributed in various regions, including 1 to 5 kb (upstream of promoter) region, exon and intron, which was higher than the proportion in 450K microarray (Supplementary Fig. S4).

### Construction of prognostic risk model

**Fig. 4:**
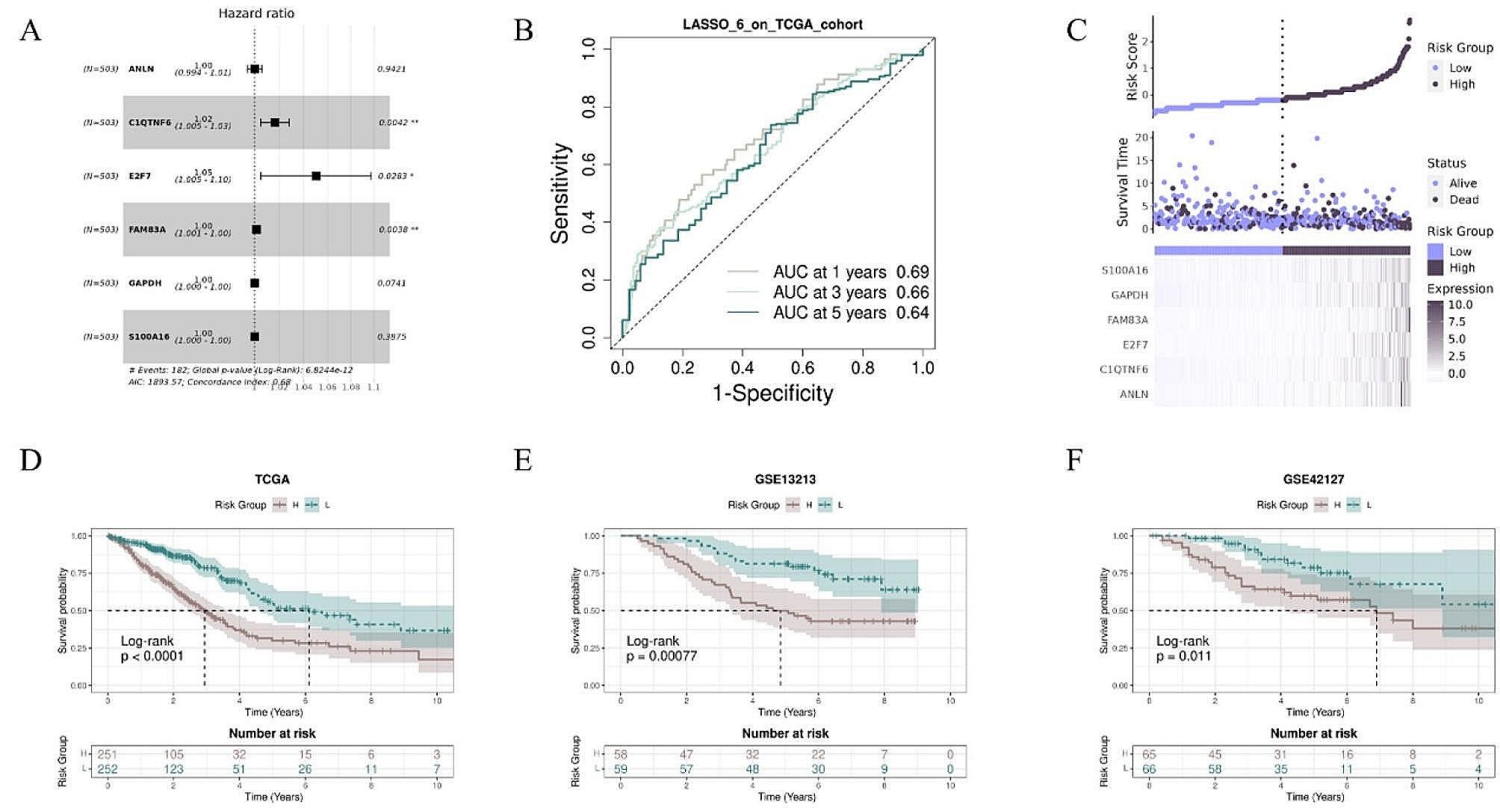
Construction of prognostic riskscore model based on methylation-driven genes **(A)** Forest plot showing 6 genes of prognostic risk model. **(B)** ROC curves for 1-, 3- and 5-year survival in the TCGA cohort. **(C)** Riskplot of TCGA patients and expression of 6 model genes in high- risk (purple) and low-risk (black) groups. **(D)** KM curves for the model in TCGA-LUAD, GSE13213,GSE42127 cohort. Patients in the high-risk group (brown) experienced a significantly shorter survival time than patients in the low-risk group (green)

To explore the impact of genes regulated by DNA methylation on prognosis, we filtered 1966 MEGs from EM-seq down to six genes to establish a prognostic risk model for overall survival (OS) in TCGA-LUAD cohort through multivariate Cox regression and LASSO model filtering. These six genes included anillin, actin binding protein (ANLN), C1q and tumor necrosis factor-related protein 6 (C1QTNF6), S100 calcium-binding protein A16 (S100A16), family with sequence similarity 83 member A (FAM83A), glyceraldehyde-3-phosphate dehydrogenase (GAPDH), and E2F transcription factor 7 (E2F7) (Fig. 4A). The areas under the tROC for 1-, 3-, and 5-year OS were estimated to be 0.69, 0.66, and 0.64 respectively in the TCGA-LUAD cohort (Fig. 4B).

The risk score was calculated for each patient in the TCGA-LUAD cohort, and high and low-risk groups were identified based on median of risk score (Fig. 4C). The riskplot illustrated that survival status in high-risk patients was poor, and the expression of the six genes was upregulated in the high-risk group. Furthermore, according to the K-M curve, the OS of the high-risk group was significantly shorter than that of the low-risk group (*p* < 0.0001). Similar results were also observed in two external datasets, GSE13213 and GSE42127 (GSE13213: *p* = 0.00077; GSE42127: *p* = 0.011, respectively) (Fig. 4D and F), suggesting a significant predictive validity for the survival of LUAD patients.

### Early-stage LUAD cellular composition differs with normal tissues

**Fig. 5:**
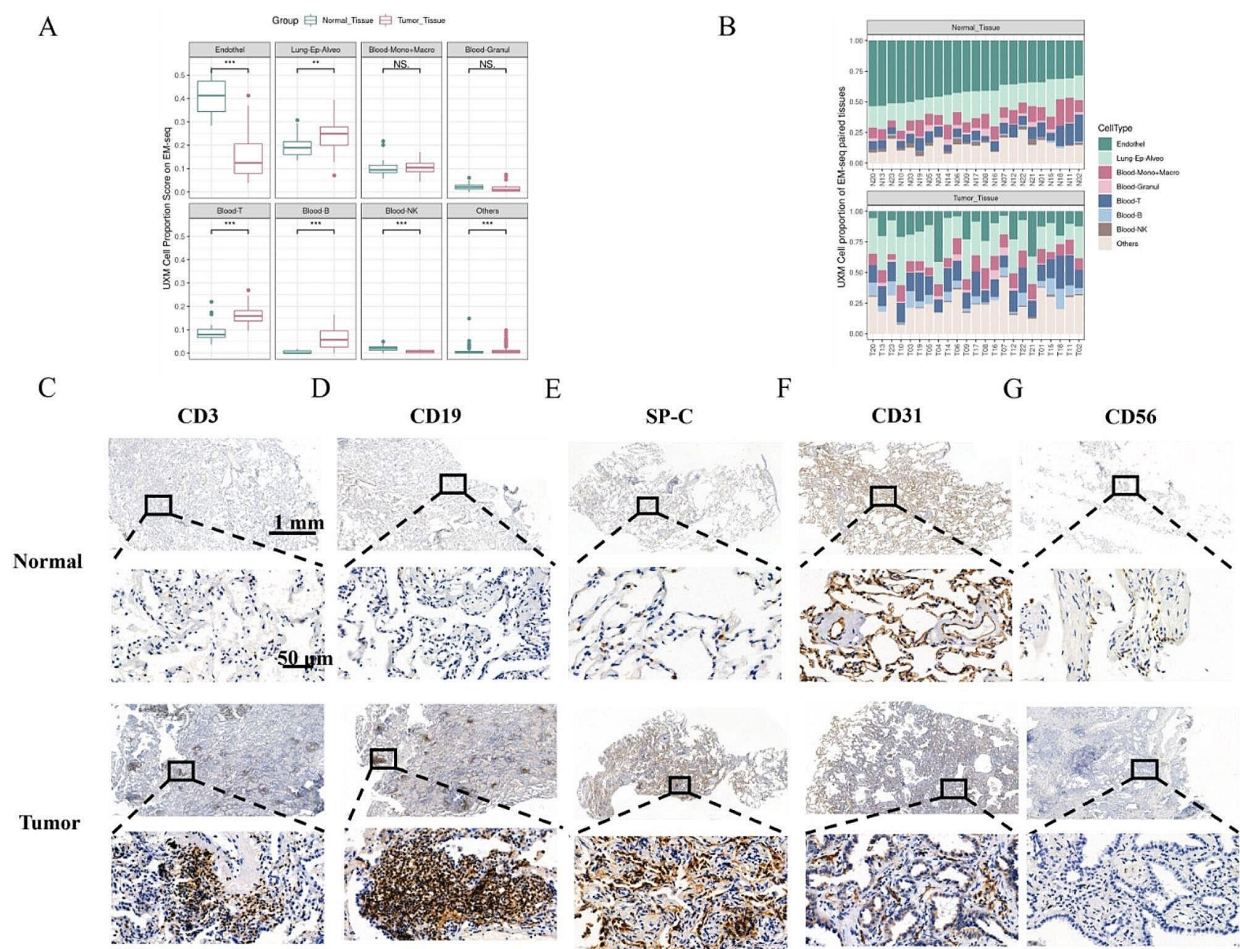
UXM analysis identified various cell populations in EM-seq and were verified by Immunohistochemistry **(A)** Cell proportion score between tumor (red) and normal (green) tissue analyzed by UXM. **(B)** The cellular composition of each tumor and normal tissue identified by UXM. **(C)** Representative immunohistochemistry image shows that CD3、CD19、SP-C were highly expressed in NSCLC tumor tissues **(C-E)**; CD31were highly expressed in normal lung tissue **(F)**; CD56 were weakly expressed in normal lung tissue **(G)**. (*p < 0.05, **p < 0.01, and ****p < 0.0001)

Finally, we applied UXM, a computational cell deconvolution algorithm, to analyze the EM-seq data and estimate cellular composition of each cell type from 23 pairs of early-stage LUAD. Our findings revealed that tumor had significantly higher levels of T cells (*p*<0.001), B cells (*p* < 0.001), lung alveolar epithelial cells (*p* < 0.01); and significantly lower levels of endothelial cells (*p* < 0.001) and NK cells (*p* < 0.001; Fig. 5A). There was no statistical difference in the ratio of monocytes, macrophages and others (such as eosinophils and basophilic granulocyte) between tumor and normal tissues. The identification and characterization of cell types for each sample were also performed (Fig. 5B). To assess the effectiveness of cell deconvolution, CD3, CD19, CD56, CD31, and SP-C were selected as markers for T cells, B cells, Natural Killer (NK) cells, endothelial cells, and lung alveolar epithelial cells, respectively. Immunohistochemistry was performed on several LUAD and paired normal FFPE tissues from the 23 paired samples. The staining area and intensity of CD3, CD19, and SP-C were higher in tumor tissues (Fig. 5C and D). Normal lung alveoli rich in capillaries were strongly stained for CD31 (Fig. 5F), and the staining intensity of CD56 was slightly higher in normal tissues (Fig. 5G). The immunohistochemical patterns of all cell types were relatively consistent with cell deconvolution results, indicating that methylation sequencing data through cell deconvolution can effectively and precisely reflect true situation of cellular composition in early-stage LUAD.

## Discussion

The overall genomic DNA hypomethylation and gene-specific DNA hypermethylation or hypomethylation are common epigenetic phenomena observed in the development of tumors [25], which is consistent with our findings in this study [26, 27]. Advancements in sequencing technologies have provided a more comprehensive identification of potential epigenetic landscapes, contributing to a better understanding of the mechanisms driving the occurrence and development of LUAD. To the best of our knowledge, our study represents the first application of EM-seq for detecting DNA methylation in early-stage LUAD. As a novel whole-genome DNA methylation sequencing method, EM-seq effectively retains methylation information in the template DNA [28]. This unique technical advantage provides new insights into various aspects of early-stage LUAD.

Currently, numerous studies have indicated that 450K microarray only reveals a limited number of methylation sites, with many of them located in the promoter region [29, 30]. In contrast, EM-seq provides higher resolution and captures a greater number of CpGs, thus identifying more DMRs. In EM-seq, except some DMRs located in promoter are also discovered, more DMRs distributed in non-transcription start site regions such as exon, intron, and intergenic regions can be identified, which are annotated to non-coding genes without encoding proteins [31–33]. They may function as cis-regulatory elements such as enhancers and silencers, or as transcribed non-coding RNA to exert regulatory functions [34–36]. For example, MIR647, MIR99AHG, and LINC00472, identified in our differentially methylated analysis, could regulate the progression of oral squamous cell carcinoma, prostate cancer, and lung adenocarcinoma by promoting cell proliferation, migration, epithelial-mesenchymal transition, and other processes [37–40]. Although some genes annotated by EM-seq DMRs currently have no research revealing their biological functions, it does not seem to affect their potential as candidate biomarkers for tumor diagnosis because base sequences have been detected. Undoubtedly it would broaden selection of biomarkers. However, further investigation and validation in future studies are necessary.

While it is generally believed that gene expression is silenced when DNA methylation occurs in promoter region [31, 41, 42], recent research has shown that hypermethylation in promoter does not always lead to gene suppression. For instance, studies have demonstrated that hypermethylation of certain promoters can actually activate gene transcription and translation. For example, the baculoviral IAP repeat containing 5(BIRC5) gene, which codes for the Survivin protein involved in both apoptosis and cell cycle regulation, has been found to be highly methylated in endometrial cancer samples, yet its high expression is correlated with hypermethylation. Moreover, demethylation using decitabine, in combination with the binding inhibitory factor P53, has been shown to suppress the tumor-promoting effects of BIRC5 [43]. In addition to transcriptional activation associated with hypermethylation in promoter, an unconventional perspective has emerged, suggesting that hypermethylation in gene body regions could also promote gene expression. One alternative mechanism is related to the synergistic regulation of transcription by histone methylation. Histone H3K4, H3K36, and H3K79 are generally regarded as markers of transcriptional activity, while H3K36me3 methylation can specifically bind to the PWWP domain of DNA methyltransferase 3(DNMT3) on gene body, promoting the establishment and maintenance of DNA methylation, and mediating transcriptional activation [44–46].While these “non-mainstream” studies are still relatively niche, potential molecular markers of this type may be found within the “Hyper-up” or “Hypo-down” groups in our study, especially in sites distributed across exons and introns. Further studies of “Hyper-up” or “Hypo-down” will enrich the regulating mechanism between epigenetic modification and gene expression.

The study primarily focused on early-stage LUAD. Both the 450 K microarray and EM-seq methods identified several well-known signaling pathways, such as the PI3K-Akt signaling pathway, cAMP signaling pathway, and Cell adhesion molecules. The PI3K-Akt signaling pathway regulates tumor cell proliferation and inhibits apoptosis [47–49] in various types of tumors, and is associated with lung cancer lymph node micrometastasis [50]. Similarly, cAMP, as a second messenger in the body, directly regulates cell metabolism, growth, and apoptosis, thereby promoting tumor progression [51, 52]. Additionally, while cell adhesion molecules are often linked to tumor metastasis, it is possible that cancer cells have already begun to disseminate or even micro-metastasize in the early stages, as observed in early breast cancer patients with metastatic tumor cells in the bone marrow [53, 54]. Therefore, cadherins, integrins, and other cell adhesion factors are likely to play a role in the early stages of lung cancer [55]. These signaling pathways, enriched in both EM-seq and 450 K microarray, appear to be consistent with the mechanisms of early-stage lung cancer. Common signal pathways involved with advanced lung cancer, were epidermal growth factor receptor/Kirsten rat sarcoma/anaplastic lymphoma kinase (EGFR/KRAS/ALK) and C-X-C motif chemokine ligand 12/C-X-C motif chemokine receptor 4 (CXCL12/CXCR4). EGFR mutations were found to be more common in multiple metastases compared to single metastasis (24/40 vs. 12/42, respectively, *p* = 0.004) [56], and EGFR + tumors were also associated with more frequent pleural (24.1% vs. 37.5%) and bone metastasis (31.5% vs. 53.8%) [57]. Additionally, CXCL12/CXCR4 is believed to be involved in angiogenesis in lung cancer [58], and high expression of CXCR4 has been significantly associated with bone metastasis [59]. These signaling pathways closely related to advanced lung cancer metastasis were not enriched in our KEGG analysis. Therefore, some signaling pathways shown in our article, may provide promising potential for revealing tumorigenesis or preventing progression to advanced-stage cancer, which are likely regulated by DNA methylation in early-stage LUAD.

The prognostic risk model based on six DMGs in our study, namely FAM83A, S100A16, E2F7, ANLN, C1QTNF6 and GAPDH, suggests that they might reveal causes of the cancer development and tumorigenesis in LUAD. FAM83A was found to be highly expressed in lung cancer, breast cancer, and pancreatic cancer [60–62].Its high expression was negatively correlated with methylation levels and predicted poor prognosis in LUAD patients [60]. It potentially promotes lung cancer progression, migration, and EMT processes through the PI3K/AKT/mTOR pathway [63] and Hippo signaling pathway [64]. S100A16 was also identified as a promising biomarker in gastric cancer early diagnosis and prediction of metastasis [65], with its high expression being associated with poor prognosis in lung cancer [66]. E2F7 and ANLN, were both involved in the cell cycle process and associated with cell proliferation in malignancy lung cancer [67–70]. C1QTNF6 had been found to be upregulated in stage I lung cancer tissue and regulated by MIR-29 A-3P to promote proliferation [71]. What’s more, inhibition of C1QTNF6 could attenuated cell migration, invasion and promoted apoptosis in non-small cell lung cancer [72]. Lastly, an uncommon prognostic gene - GAPDH, was traditionally known as a housekeeping gene. However, studies have shown that GAPDH, GUSB, and β-2 M were detected using qRT-PCR in 20 cases of lung cancer tissue and adjacent normal tissue, and the largest differences within and between groups were found in GAPDH [73]. Therefore, GAPDH seems not to be suitable as the housekeeping gene for lung cancer experiments. In fact, GAPDH can act as a key enzyme in cancer cells with the Warburg effect, responsible for controlling the rate of glucose processing [74, 75]. Additionally, GAPDH is also a Ferroptosis-Related Marker that can influence the immune microenvironment in LUAD [76]. I The six DMGs would be likely associated with key processes in lung cancer development, which aligns with their contribution to poor prognosis. However, currently, only the expression of FAM83A has been reported to be regulated by DNA methylation in tumors. Further experimental research is warranted to figure out the impact of methylation-regulated characteristic DMGs on the progression and survival of lung cancer.

Not only single-cell sequencing, but DNA methylation sequencing could accurately distinguish composition of cell type. The effective distinction of molecular and immunologic phenotypes in the early-stage of LUAD can guide the use of targeted therapy or immunotherapies. Studies now suggest that AT2 cells are the genetic origin of lung adenocarcinoma [77], hence SP-C is strongly enriched in lung cancer tissue. Additionally, research has also indicated the activation of adaptive immune responses during the progression of LUAD, manifested by the gradual enrichment of T cells and B cells, and a decrease in NK cells and granulocytes [78]. Perhaps NK cells are more abundant in small cell lung cancer (neuroendocrine tumors) [79, 80]. Our results also show a lower proportion of endothelial cells in LUAD compared to normal tissues, which is similar to a study on early diagnosis of lung cancer using single-cell transcriptomics combined with lipid metabolism. The study reported that compared to healthy lung tissue, the percentage of T lymphocytes and B cells increased in lung cancer tissue, while the percentage of single endothelial cells decreased [81]. Maybe owing to the hypervascular nature of lungs, endothelial cells were less abundant in the tumor [82]. Although the cost is relatively low, the accuracy of distinguishment of cell types using DNA methylation sequencing data through UXM algorithm appears to be satisfactory. Methylation profiles revealed by EM-seq provide insights into the cellular composition and immune environment of early-stage LUAD, offering potential theoretical basis for understanding its biological mechanisms and future early targeted interventions.

There are still some limitations in this study. Firstly, the relatively low number of samples enrolled for sequencing indicates that it only serves as exploratory research. Secondly, transcriptome sequencing was not performed on the same batch of samples. We utilized data from the TCGA LUAD RNA seq data to determine mRNA expression, which may introduce some errors to our results。.

## Conclusion

To our knowledge, this is the first study to assess genome-wide methylation in early-stage LUAD using EM-seq technology, and comprehensive applications have been conducted. In addition to confirming that EM-seq can detect a greater number of DMRs and identifying a large number of differentially MEGs that differ from the traditional “hypermethylated-downregulated” type and are located in non-promoter regions, we further integrated key MEGs to construct a prognostic model consisting of six MEGs. After validation in external databases, it showed promising predictive potential of OS. Finally, we performed cell type composition analysis of DNA methylation data using UXM cell deconvolution, achieving satisfactory accuracy in both early-stage LUAD and normal tissues. Therefore, based on the current research results, we can gain a better understanding of the epigenetic regulatory mechanisms of early-stage LUAD genome, providing potential for early diagnosis and prognosis assessment.

### Electronic supplementary material

Below is the link to the electronic supplementary material.

Supplementary Material 1

Supplementary Material 2

Supplementary Material 3

## Data Availability

Methylation data (single-site beta value) and RNA-seq data (expression count) with clinical information from TCGA program were downloaded from Genomic Data Commons data portal (https://portal.gdc.cancer.gov/). 15 patients from TCGA-LUAD cohort with both primary tumor as well as solid normal tissue samples and without any treatment or therapy were selected for our study as a comparison of our in-house methylation dataset. The clinical features of the 503 patients used to train the prognostic model, which includes 270 stage I LUAD patients. GSE42127 and GSE13213 dataset from Gene Expression Omnibus (GEO) were used as the validation data, which contained 87 and 79 stage I LUAD patients (Supplementary: Table S1).

## Abbreviations

AJCC: American Joint Committee on Cancer
ALK: Anaplastic lymphoma kinase
ANLN: Anillin β-2 M:β2-Microglobulin
BIRC5: Baculoviral IAP repeat containing 5
C1QTNF6: C1q and tumor necrosis factor-related protein 6
cAmp: cyclic AMP
C-index: concordance index
CXCL12: C-X-C motif chemokine ligand 12
CXCR4: C-X-C motif chemokine receptor
CpG: cytosine-phosphate-guanine
DEG: differentially expressed genes
DMG: differentially methylated genes
DMR: differentially methylated region
E2F7: E2F transcription factor 7
EM-seq: Enzymatic methyl sequencing
EGFR: epidermal growth factor receptor
FAM83A: family with sequence similarity 83 member A
FFPE: Formalin-Fixed Paraffin-Embedded
GAPDH: glyceraldehyde-3-phosphate dehydrogenase
GEO: Gene Expression Omnibus
GUSB: β-glucuronidase
GO: Gene Ontology
IHC: Immunohistochemistry
KEGG: Kyoto Encyclopedia of Genes and Genomes
K-M: Kaplan-Meier
KRAS: Kirsten rat sarcoma
LUAD: lung adenocarcinoma
MEG: methylated-expressed gene
NK: Natural Killer
PI3K: Phosphatidylinositide 3-kinases
S100A16: S100 calcium-binding protein A16
SP-C: pulmonary-associated surfactant protein C
TCGA: The Cancer Genome Atlas
tROC: time-dependent receiver operating characteristic
TSG: tumor suppressor gene
