# Supplementary material for: Novel genome-wide DNA methylation profiling reveals distinct epigenetic landscape, prognostic model and cellular composition of early-stage lung adenocarcinoma
Source: J Transl Med. 2024 May 6;22:428. doi: 10.1186/s12967-024-05146-2 (PMC11075300; doi:10.1186/s12967-024-05146-2)
Supplement: Supplementary file 1 — Supplementary Material 1 [file 12967_2024_5146_MOESM1_ESM.docx]

**Supplementary Figs**.


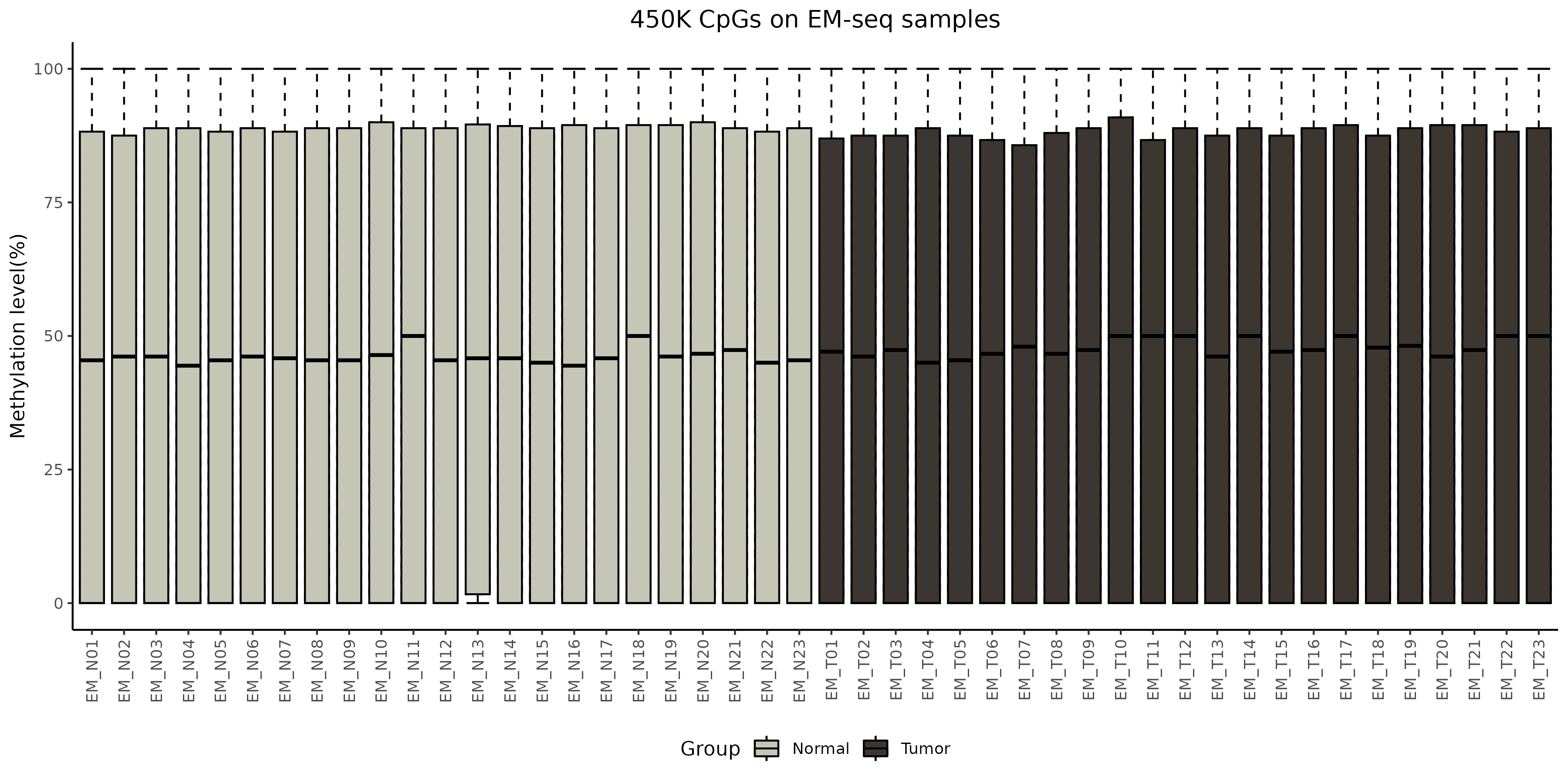


**Supplementary Fig. S1. The methylation level of 450K covered CpG sites on EM-seq samples.**

The CpG sites covered in the 450K microarray were selected to evaluate their methylation levels in each EM-seq sample, and the upper quartile, median, and lower quartile were shown through box plot.


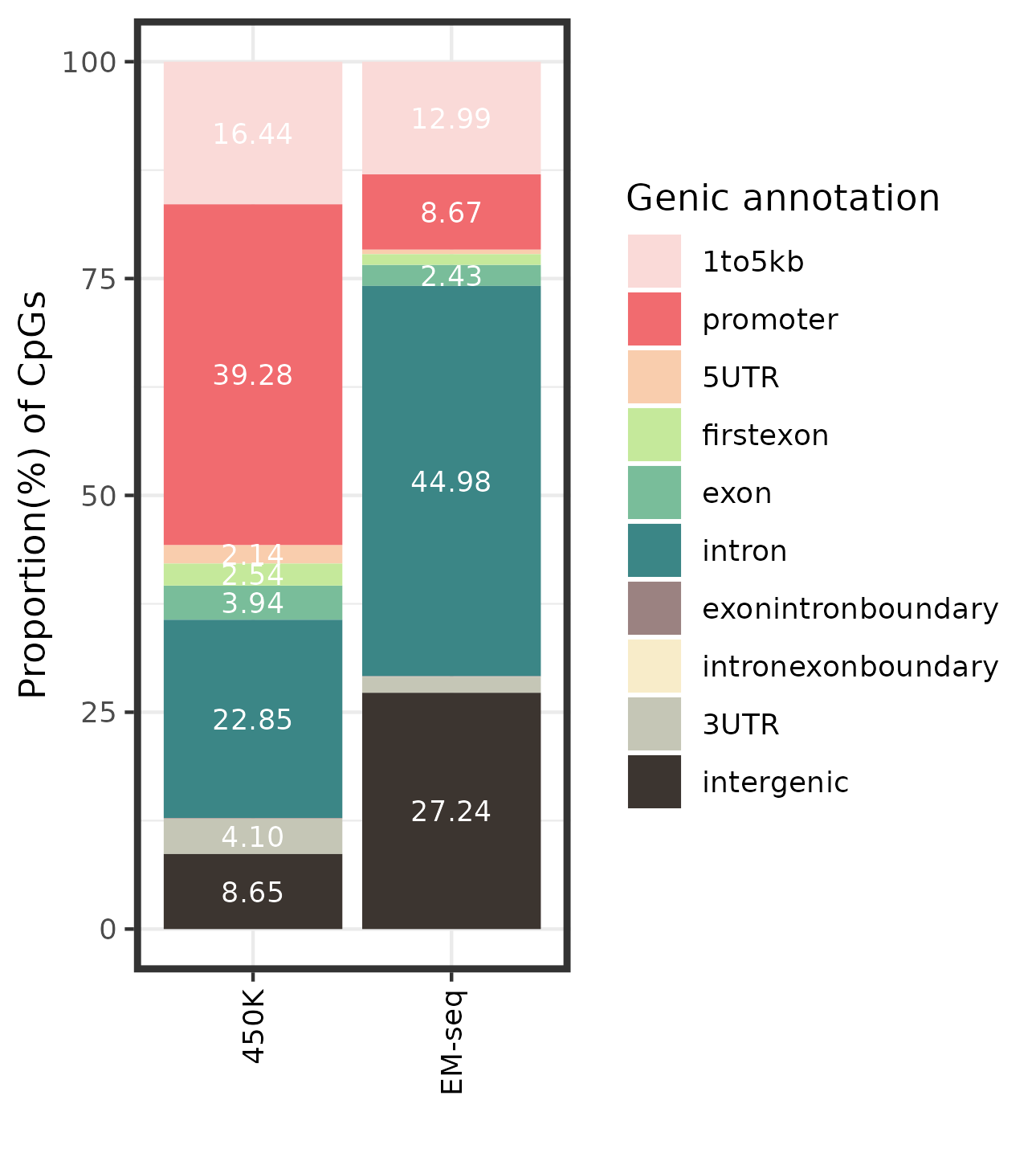


**Supplementary Fig. S2. The proportion of CpGs distributed in various genic annotation of 450K and EM-seq.**

Through genic annotation, distribution of CpGs of 450K (left) and EM-seq(right) was shown.


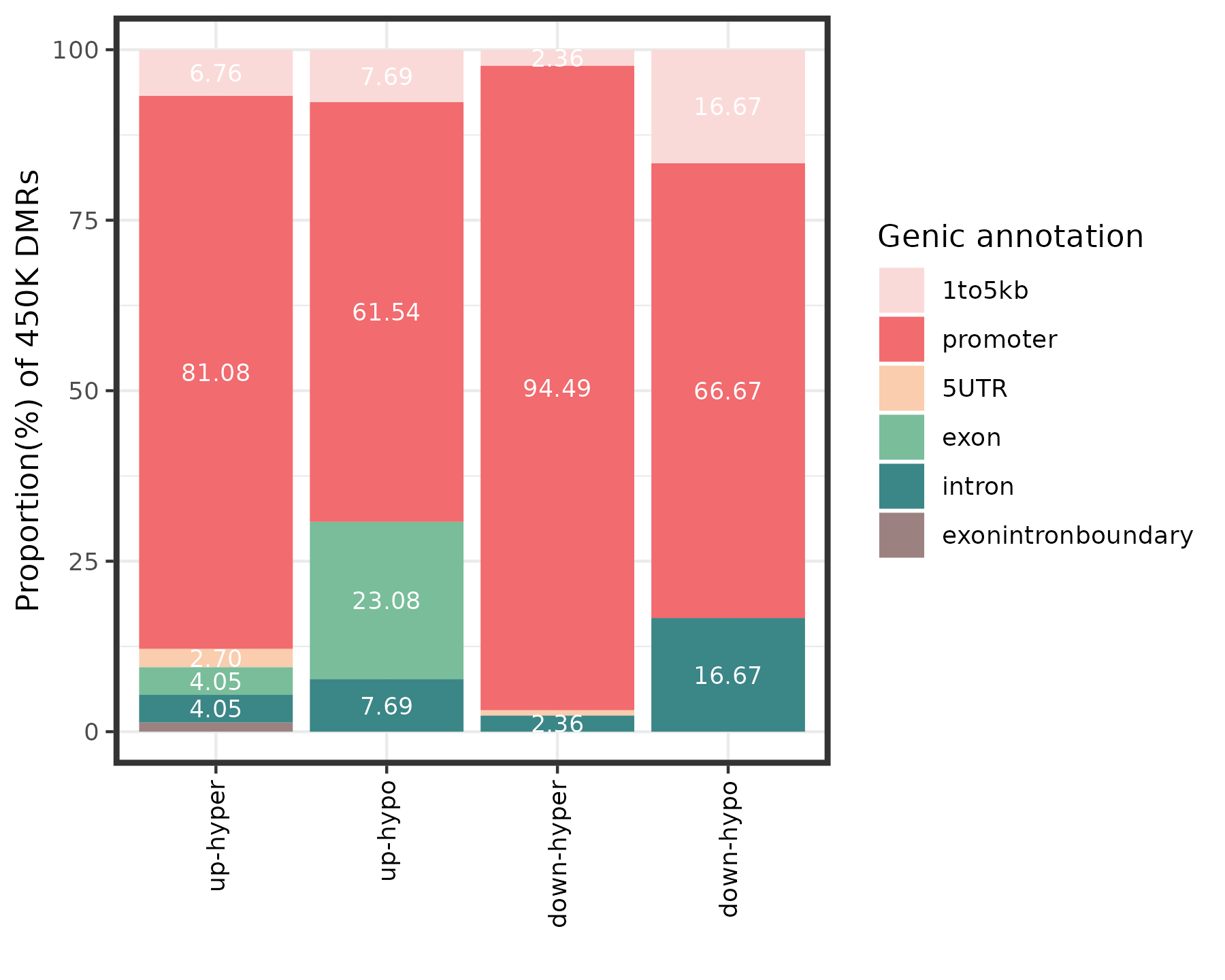


**Supplementary Fig. S3. The proportion of 450K DMRs distributed in various genic annotation associated with expression.**

Based on two types of DMR (hyper-DMR and hypo-DMR) in 450K and their corresponding gene expression (upregulate and downregulate), they were categorized into four subgroups："up-hyper" for the upregulated and hypermethylated genes; "up-hypo" for the downregulated and hypermethylated genes; "down-hyper" for the downregulated and hypermethylated genes and "down-hypo" for the downregulated and hypomethylated genes, followed by an analysis of the distribution of DMR on the genome for each subgroup.


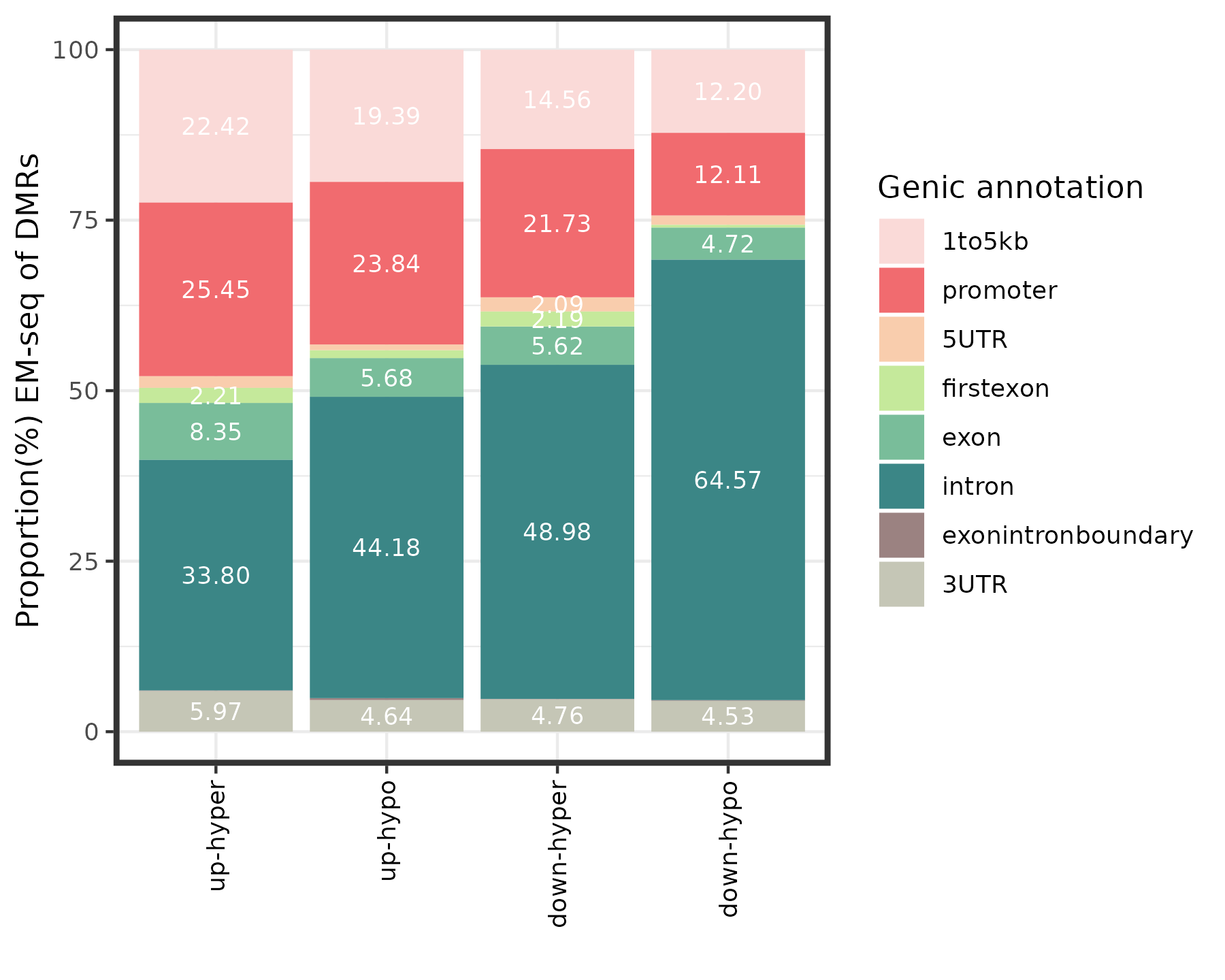


**Supplementary Fig. S4. The proportion of EM-seq DMRs distributed in various genic annotation associated with expression.**

Based on two types of DMR (hyper-DMR and hypo-DMR) in EM-seq and their corresponding gene expression (upregulate and downregulate), they were categorized into four subgroups："up-hyper" for the upregulated and hypermethylated genes; "up-hypo" for the downregulated and hypermethylated genes; "down-hyper" for the downregulated and hypermethylated genes and "down-hypo" for the downregulated and hypomethylated genes, followed by an analysis of the distribution of DMR on the genome for each subgroup.
