# Supplementary material for: Novel genome-wide DNA methylation profiling reveals distinct epigenetic landscape, prognostic model and cellular composition of early-stage lung adenocarcinoma
Source: J Transl Med. 2024 May 6;22:428. doi: 10.1186/s12967-024-05146-2 (PMC11075300; doi:10.1186/s12967-024-05146-2)
Supplement: Supplementary file 3 — Supplementary Material 3 [file 12967_2024_5146_MOESM3_ESM.docx]

Dr. Francesco Marincola

Editor-in-Chief

Journal of Translational Medicine

January 13, 2024

Dear Dr. Francesco Marincola,

We would like to submit our manuscript entitled “**Novel genome-wide DNA methylation profiling reveals distinct epigenetic landscape, prognostic models and cellular composition of early-stage Lung Adenocarcinoma**” for possible publication as an original research article in ***Journal of Translational Medicine***.

Lung adenocarcinoma (LUAD) has been a leading cause of cancer-related mortality worldwide. Early intervention can significantly improve prognosis. DNA methylation could occur in the early stage of tumor. Comprehensive understanding the epigenetic landscape of early-stage LUAD is crucial in understanding tumorigenesis.

Here, we report that Enzymatic methyl sequencing (EM-seq) was performed on 23 tumors and paired normal tissue. Methylome analysis of EM-seq comparing tumor and normal identified 25 million cytosine-phosphate-guanine (CpG) sites and 30187 differential methylated regions (DMR) with a greater number of untraditional types. By integrating the differential methylated genes (DMGs) with LUAD-related differentially expressed genes (DEGs) from the TCGA database, we constructed prognostic model based on six differentially methylated-expressed genes (MEGs) and verified our prognostic model in GSE13213 and GSE42127 dataset. Finally, deconvolution of EM-seq methylation data identified cell type composition of LUAD.

We believe the present work will be interesting to the general readership of ***Journal of Translational Medicine***, as reasons listed below:

1) We reveal the novel epigenetic landscape and EM-seq is the first application in early-stage LUAD.
2) We find huge number of DMRs including untraditional type in early-stage LUAD by EM-seq.
3) We construct a new prognostic model associated with DNA methylation in early-stage LUAD by EM-seq.

All authors have read and approved the submission of the manuscript, which has not been published and is not being considered for publication elsewhere in whole or part in any language. The authors have no conflicts of interest to declare.

We hope you will recognize these novel findings of our manuscript and send it for peer review. Thank you lor your consideration.

Sincerely yours,

Qingdong Cao, M.D.

Professor in Thoracic Surgery

Province Key Department of Thoracic Surgery in China

No. 52, E Meihua Rd, Xiangzhou District

Zhuhai, Guangdong, China 519000

Email: caoqd@mail.sysu.edu.cn
